# Use of artificial intelligence for outcome assessment in pediatric rehabilitation: a scoping review

**DOI:** 10.1186/s12984-026-01927-6

**Published:** 2026-03-05

**Authors:** Neda Naghdi, Adam Farhat, Michael Amara, Eleni Philippopoulos, Noémi Dahan-Oliel

**Affiliations:** 1 Shriners Hospital for Children, Montreal, Canada; 2 School of Physical and Occupational Therapy, Faculty of Medicine and Health Sciences, McGill University, Montreal, Canada; 3 Schulich Library of Physical Sciences, Life Sciences, and Engineering, McGill University, Montreal, Canada

**Keywords:** Artificial intelligence, Machine learning, Outcome assessment, Pediatric rehabilitation

## Abstract

**Background:**

Artificial intelligence (AI) is increasingly being applied in healthcare, with growing potential to enhance rehabilitation. In pediatric rehabilitation, traditional outcome measures are resource-intensive, time-consuming, and prone to variability, limiting their scalability. AI offers opportunities to automate, standardize, and expand access to outcome assessment. However, the scope, methodological rigor, and clinical utility of AI applications in this field remain unclear. This scoping review examined how AI has been applied to pediatric rehabilitation outcome assessment, focusing on populations studied, AI methods and models applied, outcome domains, stage of implementation, and reported limitations.

**Methods:**

A scoping review was conducted in accordance with PRISMA-ScR guidelines MEDLINE (Ovid), CINAHL (EBSCOhost), Embase (Ovid), and IEEE Xplore were searched from database inception to June 9, 2025, yielding 11,370 records; 51 studies met the eligibility criteria and were included. Study selection followed the Population, Concept, and Context (PCC) framework. Screening and data extraction were performed in Covidence by three reviewers with piloting at each stage. Data were synthesized descriptively in tables and narrative summaries. Reported AI model performance was extracted as the highest metric provided in each study (≥ 90% accuracy, F1-score, sensitivity, or specificity).

**Results:**

Fifty-one studies met the inclusion criteria. Most studies were exploratory and conducted at preclinical or early pilot stages. Children with cerebral palsy were the most frequently studied population, particularly in relation to gait analysis. AI applications were predominantly focused on motor-related outcomes, including gait, movement quality, upper-limb function, and ambulation ability, while non-motor domains such as cognitive or behavioral outcomes were sparsely represented. Supervised machine learning was the most commonly used AI type, followed by neural networks and deep learning approaches, with model selection closely aligned to data modality and task requirements. AI was most often applied for classification, prediction, and automated quantification or scoring. While several studies reported high performance for specific tasks, methodological heterogeneity, limited external validation, and small sample sizes constrained comparability and clinical translation.

**Conclusion:**

AI-based outcome assessment in pediatric rehabilitation is an emerging and rapidly evolving field, with the strongest evidence to date in motor-related applications, particularly gait analysis. Current AI tools remain largely supportive and analytical, rather than integrated into real-time clinical decision-making. Future research should prioritize methodological rigor, broader representation of pediatric populations and outcome domains, feasibility and implementation studies, and explicit consideration of ethical and equity-related issues to support responsible and clinically meaningful adoption of AI in pediatric rehabilitation.

**Supplementary Information:**

The online version contains supplementary material available at 10.1186/s12984-026-01927-6.

## Background

Artificial intelligence (AI) is increasingly being applied across healthcare, including diagnostic imaging, predictive analytics, and clinical decision-making [1, 2]. In rehabilitation, AI techniques such as machine learning (ML), computer vision, and natural language processing (NLP) are being explored to support outcome assessment and therapy planning [3–5]. Pediatric rehabilitation presents unique challenges for outcome assessment due to the heterogeneity of physical, neurological, and developmental conditions and the need for developmentally appropriate measurement approaches [6, 7]. Traditional outcome measures, while clinically essential, are often time-consuming, subjective, and dependent on clinician expertise, which may limit consistency and sensitivity to change over time [5, 7]. AI-based approaches may help address these challenges. For example, computer vision can enable automated analysis of movement using video data, and ML algorithms can identify patterns in complex datasets that may not be readily detectable through human observation [5, 8]. These technologies may support more standardized, efficient, and scalable outcome assessment and facilitate remote monitoring and tele-rehabilitation for children with limited access to specialized care [8].

Despite growing interest in AI within rehabilitation, its application to pediatric outcome assessment remains limited. Most existing studies focus on adult populations, where outcomes are generally more standardized and easier to model computationally [7, 9]. Pediatric rehabilitation introduces additional complexities, including developmental variability, limited normative datasets, and ethical considerations related to data collection and algorithmic decision-making [9]. Consequently, the current literature is fragmented across disciplines, and there is a lack of synthesis describing how AI is being applied specifically to outcome assessment in pediatric rehabilitation.

Key questions therefore remain unanswered, including which AI methods are being used, which pediatric populations are being studied, what outcomes are assessed, and how these approaches are being validated. Given the interdisciplinary and rapidly evolving nature of this field, a scoping review is an appropriate method to systematically map the existing evidence, clarify key concepts, and identify knowledge gaps without restricting inclusion based on study design or level of evidence [10–12]. The aim of this scoping review is to describe the use of AI in pediatric rehabilitation outcome assessment by mapping the populations studied, outcomes assessed, AI methods and models applied, stages of implementation, and reported limitations.

## Methods

### Protocol and reporting

This scoping review was conducted in accordance with the Arksey and O’Malley framework [11], refined by Levac et al. [10], and guided by Joanna Briggs Institute methodology [13]. Reporting followed the PRISMA-ScR checklist [14]. The review protocol, including eligibility criteria, search strategy, and data charting plan, was developed a priori and refined after pilot testing.

### Research question

The review was designed to address the following question:

"What is known about the use of artificial intelligence in outcome assessment for pediatric rehabilitation, specifically regarding the populations studied, the types of outcomes assessed, the AI methods and models applied, stage of implementation, and reported limitations?"

The question was structured using the Population–Concept–Context (PCC) framework [15].

### Eligibility criteria

Eligibility criteria were defined a priori using the PCC framework and are summarized in Table 1. Studies applying AI to outcome assessment in pediatric rehabilitation were included, regardless of the anatomical region assessed. Outcomes related to upper extremity function, lower extremity function, or whole-body movement were all eligible, provided the primary focus was outcome assessment rather than intervention delivery.

**Table 1:** Eligibility criteria for included studies based on the PCC framework

PCC framework	Inclusion criteria	Exclusion criteria
Population	Children and adolescents (≤ 18 years) with rehabilitation needs (e.g., cerebral palsy, autism, muscular dystrophy). Mixed-age studies included if pediatric data provided.	Studies with adult-only populations or where pediatric results were not provided.
Concept	AI/ML used for outcome assessment of motor, cognitive, functional, sensory, and/or behavioral areas.	AI for diagnosis, screening, treatment optimization without outcome assessment.
Context	Studies conducted in rehabilitation or healthcare settings (e.g., hospital, outpatient clinic, home-based rehabilitation, or research laboratory).	Studies outside rehabilitation contexts (e.g., education, public health).
Study Type	Primary research of any design (experimental, observational, feasibility, validation, retrospective, clinical trial). Systematic reviews/meta-analyses explicitly addressing AI outcome assessment.	Opinion papers, editorials, letters, book chapters, patents, conference abstracts without full text.
Publication	Peer-reviewed journal articles or conference proceedings published in English.	Non–peer-reviewed, non-English publications.

PCC: Population, Concept, and Context; AI: Artificial intelligence; ML: Machine learning

### Information sources and search strategy

The search strategy was developed in consultation with a health sciences librarian to ensure comprehensiveness and precision. We searched four electronic databases: MEDLINE (Ovid), CINAHL (EBSCOhost), Embase (Ovid), and IEEE Xplore. Searches were run from database inception to June 9, 2025.

Search strategies combined controlled vocabulary terms (e.g., MeSH for MEDLINE, Emtree for Embase) with free-text keywords. Two concept domains were constructed: i) artificial intelligence/machine learning and ii) pediatric rehabilitation and outcome assessment. These were combined with Boolean operators, adjacency operators, and truncation symbols as appropriate for each database. To balance sensitivity and specificity, broad AI terms (e.g., “artificial intelligence,” “machine learning,” “deep learning,” “neural networks”) were paired with outcome-related terms (e.g., “assessment,” “evaluation,” “functional outcome,” “rehabilitation outcome”) and pediatric terms.

### Study selection

**Fig. 1:**
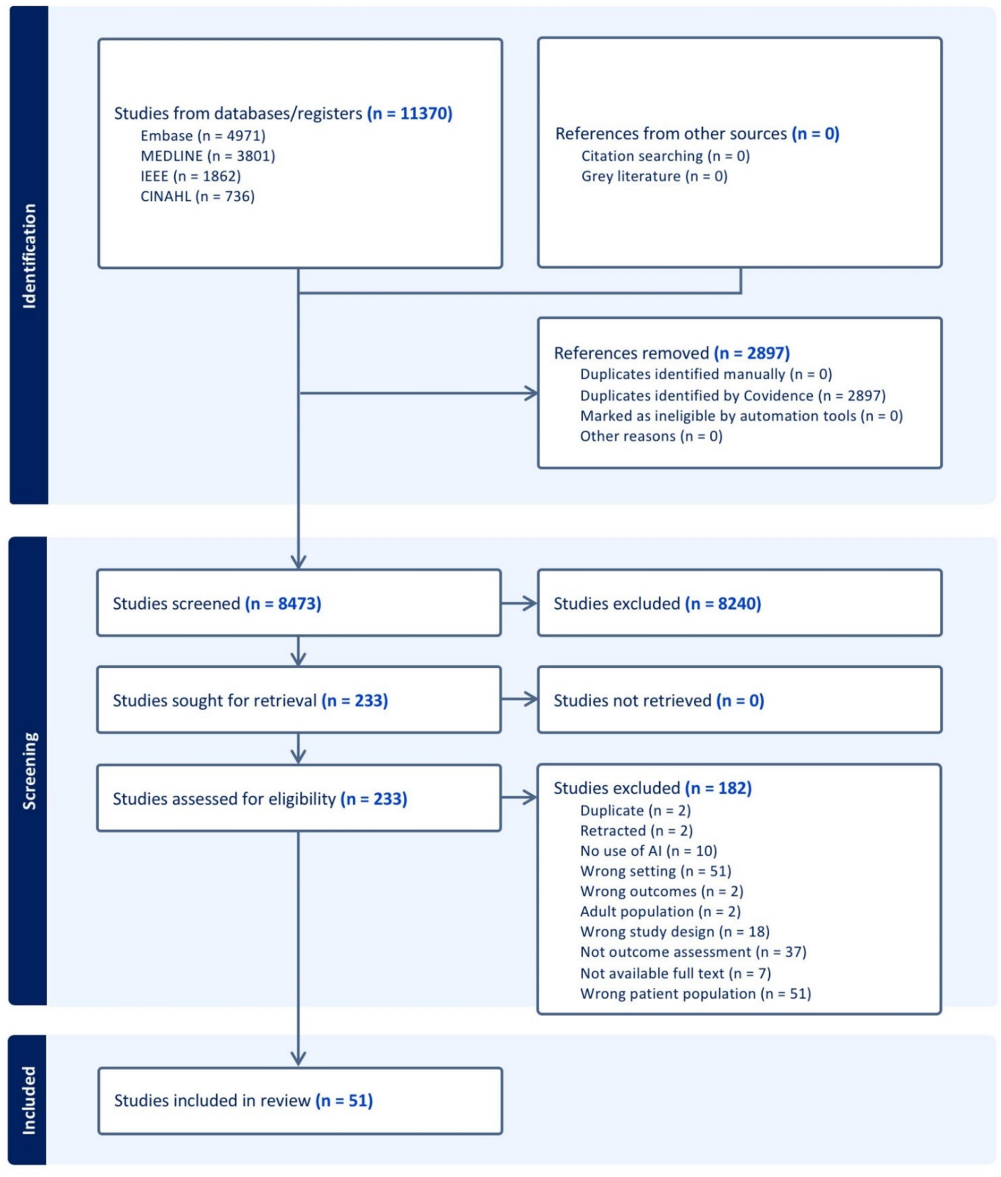
PRISMA flow chart

All search results were imported into Covidence for reference management, deduplication, and screening. After removal of 2,897 duplicates from an initial pool of 11,370 database records and three additional records identified through citation searching, 8,473 records remained for title and abstract screening. Screening and data extraction were conducted by reviewers with expertise in pediatric rehabilitation, AI-related outcomes research, and scoping review methodology, with search strategy development supported by a health sciences librarian. See Fig. 1 for the PRISMA flow diagram.

Screening was conducted using Covidence in two stages:

1. **Title and abstract screening**: Performed independently by two reviewers against the eligibility criteria. To improve reliability, a pilot of 400 records (5% of total) was screened jointly, yielding 90.75% agreement. After calibration, the remaining records were screened individually.
2. **Full-text review**: Articles that appeared potentially eligible from the title and abstract screening stage were retrieved in full and reviewed independently by three reviewers with each full-text being reviewed by two blinded reviewers. A pilot of 24 full texts (10% of total) yielded 75% agreement, prompting refinement of the inclusion criteria before continuing. Disagreements were resolved through discussion, and a fourth reviewer was available in case of unresolved conflicts.

### Data extraction and charting

Data extraction was conducted in Covidence using a standardized charting form that was developed iteratively by the review team. The form was piloted on four articles (5% of included studies), with three reviewers independently extracting data and achieving 95% agreement. Extracted data items included: bibliographic information (authors, year, country of study), study design and setting, population characteristics (sample size, age range, condition), AI/ML techniques used (algorithm AI type, specific AI model, data sources), rehabilitation domain (e.g., motor function, cognition, behavior, sensory), outcome measures assessed (classification, quantification/measurement, prediction, detection ) and, main findings (applications, reported effectiveness, ethical consideration, limitations, implications for rehabilitation practice). Performance metrics (e.g., accuracy, F1-score, AUC) were extracted as reported by the original study authors. No re-analysis or recalculation of model performance was performed. Reported metrics were derived from heterogeneous evaluation approaches, and therefore high accuracy values should be interpreted cautiously in light of methodological heterogeneity across studies. Data were charted according to five categories: population, AI methods and models outcome assessment, stage of implementation, and reported limitations.

### Data synthesis

Data were extracted using a standardized charting form developed and piloted by the review team. Extracted variables included study characteristics, population details, AI methods and models, rehabilitation domains, outcome measures, stage of implementation, and reported limitations. Data were charted across five predefined categories aligned with the review objectives.

## Results

### Search results

The search identified 11,370 records. After removal of 2,897 duplicates, 8,473 records remained for title and abstract screening. Of these, 233 full-text articles were assessed, and 51 studies met the inclusion criteria. Reasons for exclusion at the full-text stage were documented and are detailed in the PRISMA flow diagram (Fig. 1). The most common reasons included wrong setting (*n* = 51), wrong patient population (*n* = 51), no outcome assessment (*n* = 37), wrong study design (*n* = 18), and absence of AI use (*n* = 10). The selection process is presented in the PRISMA flow diagram (Fig. 1).

### Characteristics of included studies

**Fig. 2:**
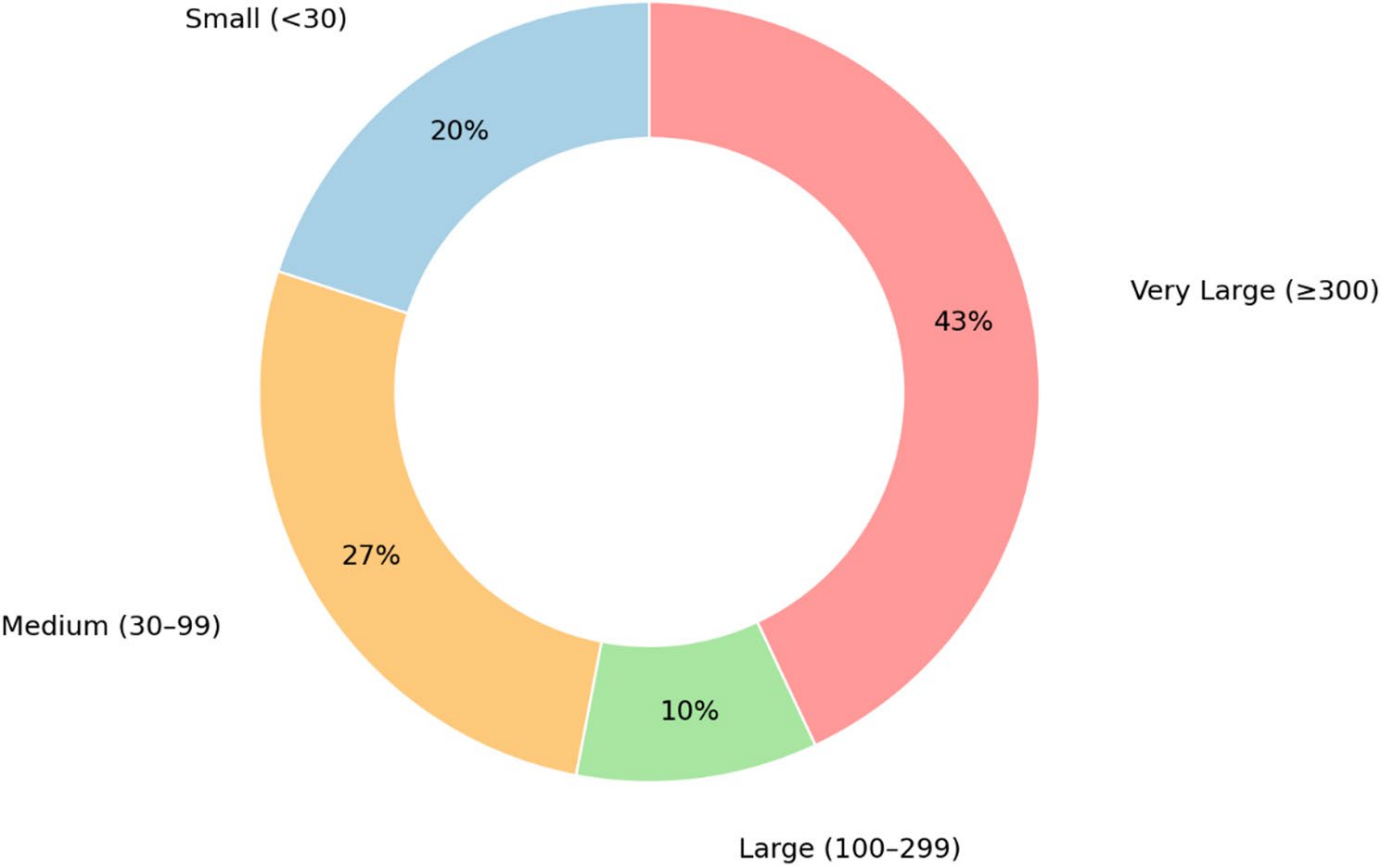
Proportion of studies by sample size category: Small (< 30), Medium (30–99), Large (100–299), Very Large (≥ 300)

The included studies varied in design, scope, and application of AI within pediatric rehabilitation. The majority were cross-sectional designs (54%), followed by cohort studies (20%). Most studies were exploratory in nature, focusing on feasibility and early validation of AI methods, with relatively few progressing to large-scale clinical implementation. Figure 2 indicates that large and very large sample sizes accounted for 53% of the studies, while small and medium-sized studies accounted for slightly less than half of the included studies.

**Fig. 3:**
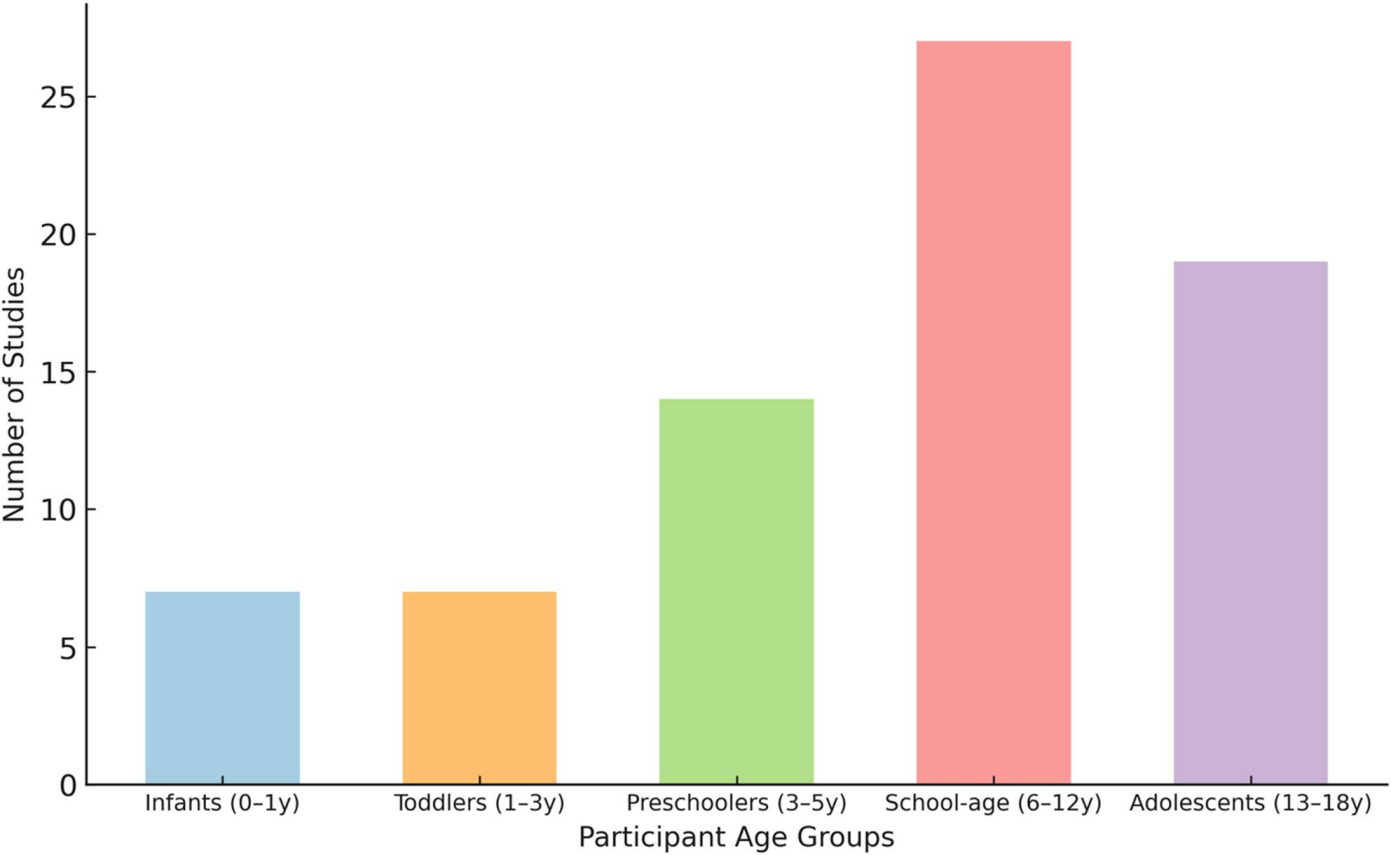
Histogram showing the distribution of participant age groups

Studies spanned multiple geographical regions, with the largest number originating from North America, Europe, and East Asia. Participant ages covered a wide pediatric range, though school aged children (6–12 years) were the most frequently represented group (Fig. 3).

### Population

The 51 included studies represented diverse pediatric populations. Children with cerebral palsy (CP) were most frequently studied, accounting for 30 studies (58.8%), particularly in relation to gait analysis (Table 2) [9, 16–20]. A smaller number of studies targeted children with other neuromotor, developmental, or medical conditions such as autism spectrum disorder, attention deficit hyperactivity disorder, juvenile idiopathic arthritis, developmental coordination disorder, developmental language disorder, spina bifida, traumatic brain injury, and Pompe disease [21–25]. Populations also included children with general neuromotor impairments, gait pathologies, and those referred for suspected developmental delay [26–30]. While CP dominated the evidence base, the diversity of diagnoses highlights the broad potential for AI-based outcome assessment across pediatric rehabilitation.

**Table 2:** Distribution of target populations across included studies

Primary population	Number of studies (*n*)	Specific condition included
Cerebral palsy (CP)	30	Spastic (uni/bilateral), dyskinetic, ataxic, mixed, GMFCS I–V
Neurodevelopmental disorders (non-CP)	7	ASD, ADHD, DLD, DCD
Mixed pediatric disability populations	5	Motor + cognitive/communication impairments
Neuromotor / neurological conditions	4	Spina bifida, Pediatric TBI, neuromuscular disorders
Other pediatric conditions	3	JIA, cleft lip/palate, HIV encephalopathy, Pompe disease
High-risk / early identification CP	2	Prematurity, neonatal brain injury

ADHD: Attention Deficit Hyperactivity Disorder; ASD: Autism Spectrum Disorder; DCD: Developmental Coordination Disorder; DLD: Developmental Language Disorder; GMFCS: Gross Motor Function Classification System; JIA: juvenile idiopathic arthritis; TBI: Traumatic Brain Injury

### Outcome categories

Primary outcomes focused on the most part on gait (16 studies), whereas other areas in which AI was used included physical activity, speech, attention, upper extremity use, self-care, functional skills and pain in the context of pediatric rehabilitation. These outcomes were categorized as classification, quantification/measurement, prediction, detection, and other exploratory outcomes based on how AI was used in each study:

#### Classification

Assignment of participants, images, signals, or clinical data to predefined categories or classes. In pediatric rehabilitation, this includes determining whether movement patterns or functional outcomes correspond to specific diagnostic, functional, or performance groups (e.g., typical vs. pathological gait).

#### Quantification/measurement

Derivation of objective numerical values representing rehabilitation-related parameters, such as joint kinematics, range of motion, spatiotemporal gait variables, or other measurable functional outcomes using AI-based methods.

#### Prediction

Use of AI models to estimate or forecast future clinical outcomes, functional progression, or response to intervention based on baseline or longitudinal data.

#### Detection

Automatic identification of the presence or occurrence of specific events or features within data streams (e.g., detection of gait events, abnormal movement patterns, or clinically relevant signals).

#### Other

Outcome assessment applications that did not align with the above categories, including study-specific, exploratory, or feasibility-related outcomes (e.g., usability, system feasibility, or novel outcome constructs).

**Fig. 4:**
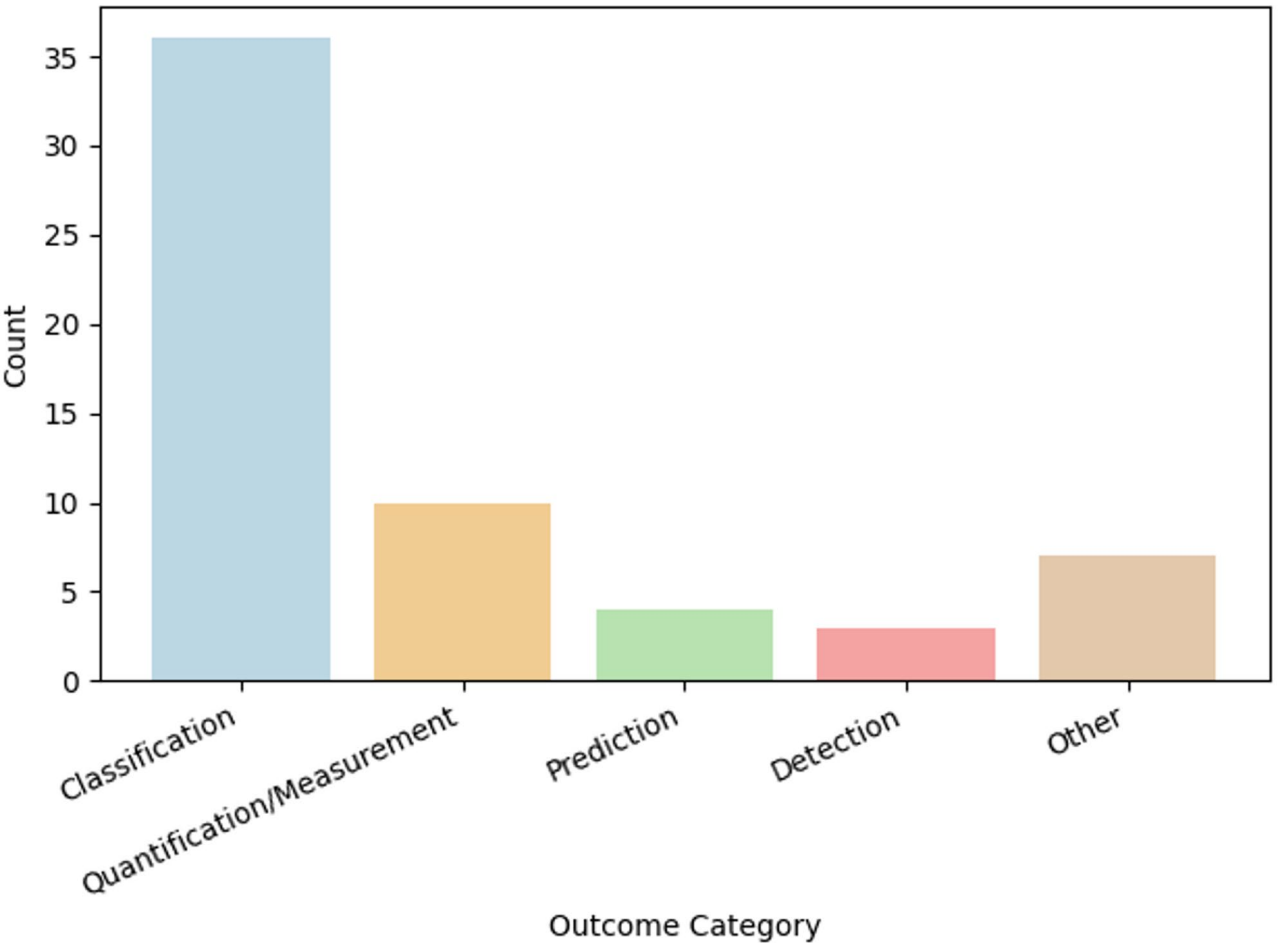
Distribution of included studies by primary outcome category

The distribution of primary outcome categories is illustrated in Fig. 4.

### AI methods applied

**Fig. 5:**
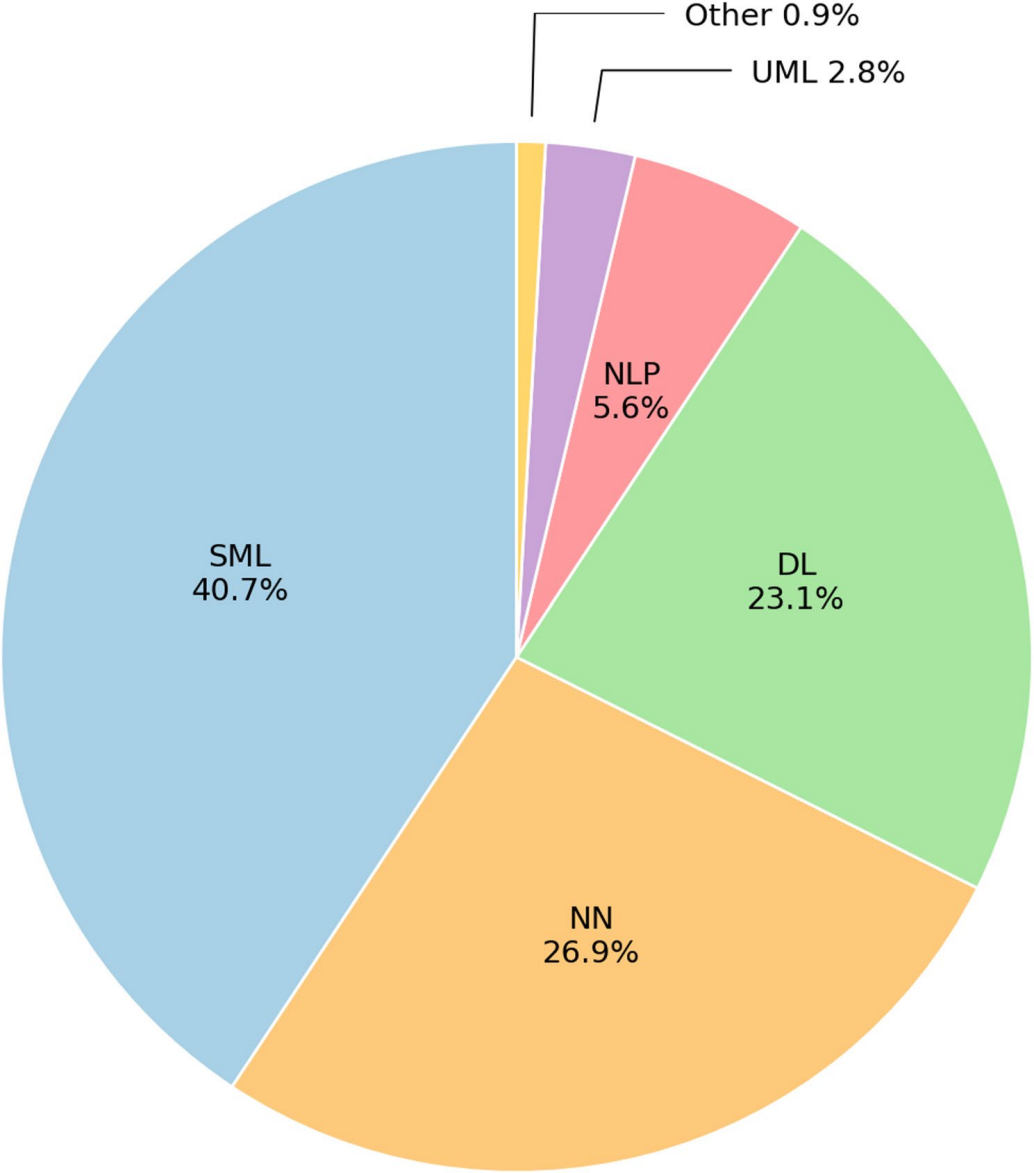
Pie chart representing the distribution of AI types in the included studies. DL: Deep Learning; NLP: Natural Language Processing; NN: Neural Network; SML: Supervised machine learning; UML: Unsupervised machine learning

Across all included studies, regardless of reported performance, supervised machine learning constituted the largest proportion of AI methods (40.7%), followed by neural networks (26.9%) and deep learning approaches (23.1%). Deep learning approaches were predominantly applied to image and video data, whereas supervised learning techniques such as support vector machines (SVMs) were more commonly used with structured clinical or sensor-derived data (Fig. 5).

When specific AI models were reported, convolutional neural networks (CNNs) were most common (*n* = 5), followed by long short-term memory (LSTM, *n* = 2), gated recurrent unit (GRU, *n* = 1), XGBoost (*n* = 1), and traditional artificial neural networks (ANN, *n* = 1) (Table 3).

**Table 3:** Counts by specific AI model

Specific AI model	Count
CNN	5
LSTM	2
GRU	1
XGBoost	1
ANN	1

CNN: Convolutional Neural Network; LSTM: Long Short-Term Memory; GRU: Gated Recurrent Unit; ANN: Artificial Neural Network

### Reported accuracy and performance

 Across studies that reported performance metrics, high accuracy was most commonly achieved using supervised machine learning, neural network, and deep learning methods. Very high performance (≥ 90% accuracy or equivalent metrics) was most commonly reported for classification and prediction tasks, particularly those involving gait phase classification, event detection, and prediction of functional or engagement-related outcomes.

When performance was examined by outcome category, supervised machine learning and deep learning approaches consistently yielded the highest reported results (Table 4). In classification outcomes, the highest reported accuracy (97%) was achieved using a supervised machine learning model, specifically a multilayer perceptron. Classification tasks primarily focused on distinguishing between clinically similar gait patterns or functional movement states, rather than assigning formal diagnostic labels [31, 32]. Prediction tasks were similarly dominated by supervised ML approaches, including support vector machine–based models, with several studies reporting performance exceeding 90%. For quantification and measurement outcomes, deep learning architectures applied to video-based analysis achieved the highest reported performance within that category. An additional exploratory outcome category related to ambulation ability was identified, in which deep learning–based video analysis approaches were applied.

While ≥ 90% accuracy was considered indicative of very high performance, the highest reported values in some outcome categories were below this threshold and are reported to reflect the best available performance within those domains. Several high-performing classification models were applied to conditions characterized by overlapping motor control features, emphasizing the relevance of AI-based approaches for differentiating between clinically similar movement patterns rather than relying solely on diagnostic labels [33, 34].

**Table 4:** Highest reported performance of AI type and model by outcome category

Outcome category	Primary outcome	AI type	Specific model	Highest reported performance	Study references
Classification	Gait phase classification (stance vs. swing) Prediction of heel-strike and toe-off event timing	Supervised ML	MLP	97.0%	Morbidoni 2021 (35)
Prediction	Prediction accuracy (macro-averaged F1 score) of AI models for classifying patient engagement during RAGR (based on self-perceived and therapist-perceived engagement levels)	Supervised ML	SVM	95.6%	Costantini 2024 (29)
Quantification/measurement	Sensitivity/specificity of AI video analysis vs. traditional BSID-II therapist assessment for “Places Pegs in” and “Blue Board” tasks Quantitative measures: total time, number of trials, successful trials, time and spatial intervals	DL	YOLOv5 (object detection for video)	86.5%	Ye 2025 (8)
Other (ambulation ability)	Ambulation ability, categorized into community ambulators, household ambulators, therapeutic ambulators, and non-ambulators.	DL	RNN implemented as a MLP	83.2%	McKernan 2025 (36)

Highest reported performance for each major outcome category, with corresponding broad AI type, specific model, and source study. Performance values represent the best reported result within each outcome category as described in the original studies and were not recalculated by the review authors. While ≥ 90% accuracy or equivalent metrics (e.g., F1-score, sensitivity, specificity) were considered indicative of very high performance, some outcome categories reported lower peak values; these are included to reflect the highest available performance within those domains.

DL: Deep Learning; MLP: Multilayer Perceptron; RAGR: Robot-Assisted Gait Rehabilitation; BSID-II: Bayley Scales of Infant Development Second Edition; YOLO: You Only Look Once (object detection model); RNN: Recurrent Neural Network; SVM: Support Vector Machine.

### Applications across functional domains

AI-based outcome assessment was applied across multiple functional domains in pediatric rehabilitation, with a strong emphasis on motor-related outcomes. Most studies focused on gross motor function, gait, posture, and movement quality, particularly in populations with CP and other neuromotor conditions. Beyond gait analysis, AI applications targeted upper-limb function, ambulation ability, activity or engagement recognition, and quantitative assessment of movement during functional tasks. These applications employed diverse data sources, including motion capture, wearable sensors, and video-based pose estimation.

In contrast, relatively few studies addressed non-motor domains such as cognitive function, sensory processing, or behavioral performance. When included, these domains were typically assessed indirectly, for example through activity classification or engagement monitoring rather than direct domain-specific outcome measures. Across these functional domains, AI methods were applied in distinct roles within outcome assessment, as summarized below.

### Roles of AI in outcome assessment

Across the included studies, AI methods were applied to outcome assessment in several distinct roles. The most common application was classification, including differentiation of gait phases, movement patterns, functional states, or patient groups based on kinematic, sensor-derived, or video-based data. Prediction tasks involved forecasting functional outcomes, engagement levels, or performance trajectories using clinical, sensor, or motion data.

AI was also frequently used for automated quantification or scoring, whereby objective outcome measures (e.g., temporal–spatial parameters, movement counts, task completion metrics) were extracted from motion capture, wearable sensors, or video recordings. In some studies, AI-based feature extraction served as an intermediate step to derive clinically relevant variables from high-dimensional data prior to classification or prediction. A smaller number of studies applied AI for monitoring performance during functional tasks, often in rehabilitation or assessment settings.

### Applications in outcome assessment

Gait analysis emerged as a central application area within pediatric rehabilitation. Sixteen studies (30%) specifically examined gait-related outcomes, of which 10 focused on CP. Other application areas included upper-limb function assessment, gross motor performance, engagement or activity recognition, ambulation ability classification, and movement quantification during functional tasks, highlighting the broad scope of AI applications across pediatric rehabilitation outcomes.

AI approaches for gait analysis ranged from marker-based motion capture to wearable sensors and video-based pose estimation, reflecting the diversity of data sources used in clinical and research settings. This distribution underscores gait as both a clinically important outcome and a technically challenging target for AI innovation, while situating it within a wider landscape of functional and movement-based applications.

### Implementation stages

**Fig. 6:**
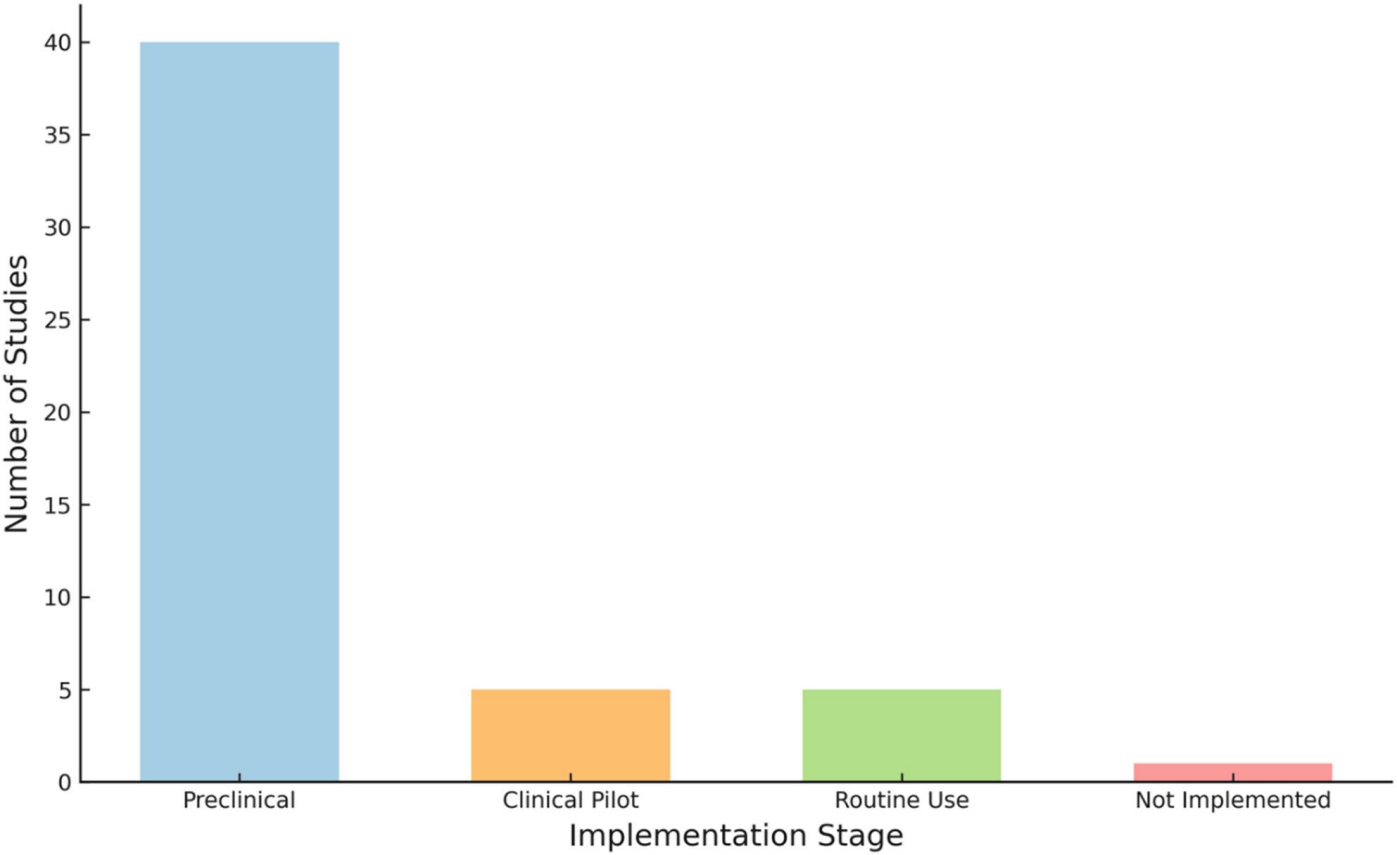
Histogram showing the number of included studies at each stage of implementation

Most studies were in early or preclinical phases, with relatively few reporting clinical pilot testing and very few progressing to routine clinical use (Fig. 6).

### Reported limitations

Consistent with scoping review methodology, no formal risk-of-bias or methodological quality appraisal was conducted. However, limitations as reported by the original studies were extracted and summarized. Commonly reported limitations included small sample sizes, lack of external validation, and challenges related to model interpretability, particularly for deep learning approaches. Several studies also reported constraints related to data availability or applicability to specific populations or settings. Notably, some studies did not report limitations. No included studies explicitly reported ethical or privacy considerations, consent or assent procedures, or data governance frameworks related to the use of AI.

## Discussion

This scoping review synthesizes how AI has been applied to pediatric rehabilitation outcome assessment. By mapping study characteristics, populations, outcome categories, AI methods, application areas, performance, and implementation stages, this review provides a structured overview of how AI is currently being developed and applied in pediatric rehabilitation contexts. Overall, the findings indicate a rapidly expanding but methodologically heterogeneous field that remains largely at an early stage of clinical translation.

### Study characteristics and maturity of the field

The predominance of exploratory and cross-sectional study designs reflects the early developmental stage of AI-based outcome assessment in pediatric rehabilitation. Most studies emphasized feasibility testing, proof-of-concept validation, or early performance evaluation rather than clinical effectiveness or implementation research [37–39]. This trend is consistent with emerging AI applications in healthcare, where methodological development and algorithm optimization often precede rigorous evaluation of clinical utility. While this approach is appropriate at an early stage, it also underscores the need for a shift toward longitudinal designs, multicenter studies, and pragmatic trials to support meaningful clinical adoption. Similarly, a scoping review on the use of digital avatars to improve virtual rehabilitation reported that further work should explore long-term effects, AI-driven personalization, and integration into health systems, in addition to ethics and privacy considerations [40].

### Populations studied

Children with CP were the most frequently studied population, particularly in relation to gait-related outcomes [41–43]. This focus likely reflects both the clinical importance of movement assessment in CP and the availability of established gait datasets that facilitate AI development. However, the limited representation of other pediatric rehabilitation populations, including children with mixed disabilities, developmental disorders, or non-neuromotor conditions, restricts the generalizability of current AI-based outcome assessment tools [36, 44]. Expanding research beyond CP will be essential to ensure that AI applications are relevant across the diverse populations encountered in pediatric rehabilitation practice.

### Outcome categories and interpretation of classification

Across the included studies, AI was applied to outcome assessment primarily through classification, prediction, and quantification or measurement tasks. Classification outcomes were the most common and were typically used to differentiate between gait phases, movement patterns, functional states, or patient groups [7, 45]. Importantly, these classification tasks were not intended to establish formal diagnoses, but rather to support differentiation of clinically similar movement presentations. This distinction is critical in pediatric rehabilitation, where overlapping motor features, particularly in early childhood, can complicate diagnosis and treatment planning [31, 32]. AI-based classification approaches therefore appear to function primarily as decision-support tools rather than diagnostic replacements.

### AI types and model selection

Supervised machine learning approaches were the most commonly applied AI type, followed by neural networks and deep learning models. This distribution reflects the nature of the data and outcomes examined in pediatric rehabilitation research. Supervised ML methods were frequently applied to structured or semi-structured data, such as spatiotemporal gait parameters, wearable sensor outputs, or clinically derived features, where smaller sample sizes and greater interpretability may be advantageous [29, 35, 46]. In contrast, deep learning models were predominantly used for image- and video-based applications, including pose estimation and automated movement quantification, where high-dimensional inputs require advanced feature extraction [8, 36]. Neural network–based models were often used for time-series analyses derived from sensor or motion capture data, occupying an intermediate methodological space [17, 27]. Collectively, these findings suggest that AI model selection is driven primarily by data modality and task requirements rather than by a universally optimal algorithm.

### Performance and interpretation of reported accuracy

High reported accuracy was most frequently observed for classification and prediction tasks, particularly in gait-related applications [7, 22, 37, 41, 45]. However, performance metrics varied across outcome categories, reflecting differences in task complexity, data quality, and evaluation methods. Many studies relied on internal validation using limited datasets, which may inflate reported performance [9, 39, 47, 48]. Consequently, high accuracy values should be interpreted as indicators of technical feasibility rather than evidence of clinical readiness [19]. Differences in reported metrics across studies further complicate direct comparison and highlight the need for standardized evaluation frameworks.

### Applications across functional domains

AI-based outcome assessment was applied predominantly to motor-related functional domains, including gait, posture, movement quality, upper-limb function, and ambulation ability [7, 8, 45, 49–51]. These domains lend themselves to objective measurement and computational modeling, which may explain their dominance in the literature. In contrast, applications targeting non-motor domains such as cognitive function, sensory processing, or behavioral performance were sparse and typically indirect, relying on activity recognition or engagement classification rather than domain-specific outcome measures [28, 39, 52–55]. This imbalance suggests that current AI research prioritizes observable motor behavior, while more complex or less directly measurable functional domains remain underexplored.

### Roles of AI in outcome assessment

Across functional domains, AI methods served distinct roles within outcome assessment. Classification and prediction were the most common applications, followed by automated quantification or scoring of movement-related parameters [18, 37, 56]. Monitoring applications were less frequently reported, and there were no included studies reported the use of closed-loop or adaptive AI systems, in which outcome assessment would directly inform real-time intervention adjustment. This pattern indicates that AI in pediatric rehabilitation is currently used primarily as an analytical and supportive tool rather than as an integrated component of therapeutic decision-making or intervention delivery.

### Gait analysis as a central application area

Gait analysis emerged as the most mature and extensively studied application area for AI-based outcome assessment. Studies employed a wide range of data sources, including marker-based motion capture, wearable sensors, and video-based pose estimation, reflecting both clinical relevance and technical feasibility [17, 27, 30, 32, 38]. While advances in gait analysis demonstrate the potential of AI to enhance objectivity and precision in outcome assessment, the strong focus on gait also highlights the need to translate methodological advances to other functional outcomes that are equally important for pediatric rehabilitation.

### Implementation stage and clinical utility

Despite promising technical performance, most AI-based outcome assessment tools identified in this scoping review remain at the preclinical or early pilot stages. In the near term, these tools are most likely to function as adjuncts to clinical assessment, supporting objective measurement, automated scoring, and pattern recognition, particularly in gait and movement analysis, rather than replacing clinician judgment. Such applications may help reduce assessment burden and improve consistency across raters.

However, practical implementation is constrained by hardware and infrastructure requirements (e.g., sensors, video capture systems, data storage), as well as challenges related to workflow integration, clinician training, cost, and interoperability with existing clinical systems [39, 52]. Without explicit attention to feasibility, usability, and scalability, many AI tools are likely to remain confined to research settings [54]. Evidence from related rehabilitation domains suggests that future studies should prioritize real-world applicability and the transfer of skills to bridge the gap between technical performance and functional clinical impact [41]. Addressing these challenges will be essential to support translation from research prototypes to clinically usable AI-enabled outcome assessment tools.

### Heterogeneity of outcomes and data sources

A major challenge identified across the included studies was the substantial heterogeneity in outcome measures, assessment tools, and data sources used for AI-based outcome assessment. Even within the same outcome category (e.g., gait or gross motor function), studies varied widely in how outcomes were defined and operationalized [34]. Assessment tools ranged from standardized clinical measures and therapist-labeled outcomes to custom task-specific metrics derived from motion capture, wearable sensors, or video analysis, limiting direct comparison of results across studies.

Heterogeneity was also evident in the types of data sources and sensing technologies employed. Studies used diverse motion capture systems, wearable inertial sensors, pressure sensors, and video-based pose estimation approaches, often with different sampling rates, sensor placements, camera viewpoints, and preprocessing pipelines [9, 17–19, 24, 27, 32, 37, 52]. These differences introduce variability in feature extraction and model inputs, which may contribute to inconsistent performance estimates across studies. In addition, outcome definitions and performance metrics were not standardized, with accuracy, F1-score, sensitivity, specificity, and task-specific measures reported inconsistently, further limiting comparability. Collectively, this methodological heterogeneity constrains synthesis across studies and complicates interpretation of reported performance. While such diversity reflects the exploratory nature of AI research in pediatric rehabilitation, it underscores the need for greater standardization of outcome definitions, reporting practices, and benchmarking frameworks to enable meaningful comparison, replication, and clinical translation.

### Limitations and ethical considerations

Methodological heterogeneity across outcome definitions, assessment tools, sensing technologies, and performance metrics limits comparability across studies and complicates synthesis. Small sample sizes, limited external validation, and challenges related to model interpretability further constrain clinical translation. Also, A formal risk-of-bias or methodological quality appraisal was not conducted, consistent with scoping review methodology, which may be a limitation. Ethical considerations were notably underreported, despite the use of sensitive pediatric data. Issues related to consent and assent, data governance, algorithmic bias, and equitable access to AI-enabled rehabilitation technologies require greater attention. Integrating ethical and governance frameworks alongside technical development will be critical to ensure responsible, inclusive, and sustainable implementation of AI-based outcome assessment in pediatric rehabilitation, consistent with recommendations from other fields [40].

## Conclusion

This scoping review provides a comprehensive overview of how AI has been applied to outcome assessment in pediatric rehabilitation, highlighting current application areas, AI methods, performance characteristics, and stages of implementation. The evidence to date demonstrates that AI-based outcome assessment has been most extensively developed for motor-related domains, particularly gait analysis, where supervised machine learning and deep learning approaches have shown promising performance in classification, prediction, and automated quantification tasks. However, the current literature remains largely exploratory, with most studies conducted at early or preclinical stages and limited evidence of routine clinical implementation. AI model selection appears to be driven primarily by data modality and task requirements rather than by a single optimal approach, underscoring the importance of aligning AI methods with clinically meaningful outcomes. Significant gaps persist in the application of AI to non-motor domains, the use of standardized outcome definitions, external validation, and integration into clinical workflows. Future research should prioritize methodological rigor, including larger and more diverse datasets, standardized evaluation frameworks, and longitudinal validation. Integrating ethical and governance frameworks alongside technical development will be critical to ensure responsible, inclusive, and sustainable implementation of AI-based outcome assessment in pediatric rehabilitation. By aligning technical development with clinical needs and responsible implementation, AI-based outcome assessment has the potential to enhance objectivity, efficiency, and consistency in pediatric rehabilitation, ultimately supporting improved clinical decision-making and patient-centered care.

## Supplementary Information

Below is the link to the electronic supplementary material.

Supplementary Material 1.

## Data Availability

The datasets used and/or analysed during the current study are available from the corresponding author on reasonable request.

## Abbreviations

AI: Artificial intelligence
ANN: Artificial neural network
ASD: Autism spectrum disorder
ADHD: Attention deficit hyperactivity disorder
BSID-II: Bayley scales of infant development, second edition
CNN: Convolutional neural network
CP: Cerebral palsy
DCD: Developmental coordination disorder
DL: Deep learning
DLD: Developmental language disorder
F1-score: Harmonic mean of precision and recall
GMFCS: Gross motor function classification system
GRU: Gated recurrent unit
JIA: Juvenile idiopathic arthritis
LSTM: Long short-term memory
ML: Machine learning
MLP: Multilayer perceptron
NN: Neural network
NLP: Natural language processing
PRISMA-ScR: Preferred reporting items for systematic reviews and meta-analyses extension for scoping reviews
RAGR: Robot-assisted gait rehabilitation
RNN: Recurrent neural network
SML: Supervised machine learning
SVM: Support vector machine
TBI: Traumatic brain injury
UML: Unsupervised machine learning
YOLO: You Only Look Once (object detection model)
