# Supplementary material for: Use of artificial intelligence for outcome assessment in pediatric rehabilitation: a scoping review
Source: J Neuroeng Rehabil. 2026 Mar 5;23:123. doi: 10.1186/s12984-026-01927-6 (PMC13072576; doi:10.1186/s12984-026-01927-6)
Supplement: Supplementary file 1 — Supplementary Material 1. [file 12984_2026_1927_MOESM1_ESM.docx]

| Database | Medline |
| --- | --- |
| Platform | Ovid |
| Limits | None |
| Date | June 9, 2025 |
| Results | 3801 |

<https://proxy.library.mcgill.ca/login?url=https://ovidsp.ovid.com/ovidweb.cgi?T=JS&NEWS=N&PAGE=main&SHAREDSEARCHID=6KEfr7qGQmelGzTfboYVqdXpJHVHnzI4Sd1IpHfuUxs6I8UyCRTmXxj0SB6sDtOmd>

Ovid MEDLINE(R) ALL <1946 to June 06, 2025>

1 exp child/ 2272476

2 adolescent/ 2333308

3 (child* or preschool or pre school or school aged or teen* or adolescen* or "young adult*" or youth* or toddler*).tw,kf,jw. 2297279

4 exp pediatrics/ 65353

5 (pediatric* or paediatric*).tw,kf,jw. 990109

6 1 or 2 or 3 or 4 or 5 4580072

7 exp Artificial Intelligence/ not exp Robotics/ 208063

8 (artificial intelligence or AI or comput* intelligence or comput* reasoning or comput* vision system* or comput* knowledge or machine intelligence or intelligence system* or intelligent system*).tw,kf. 123466

9 (machine learning or transfer learning).tw,kf. 157763

10 (dimensionality reduction* or sing* value decomposition* or linear discriminant analys*).tw,kf. 18716

11 (ensemble learning* or ensemble model aggregation* or federated learning* or reinforcement learning* or predictive learning* or representation learning* or supervised learning* or semi supervised learning* or unsupervised learning* or multiple instance learning* or support vector machine*).tw,kf. 59255

12 (sentiment analys* or sentiment classification* or opinion mining).tw,kf. 2576

13 (particle swarm or swarm intelligence or classical PSO or classical PSOs or PSO TVAC).tw,kf. 5557

14 (machine pattern* or trend analys* or machine predict*).tw,kf. 8358

15 (Computational Neural Network* or Connectionist Model* or neural network* or computer neural network* or perceptron*).tw,kf. 136500

16 (deep learning or hierarchical learning or autoencoder* or large language model* or natural language processing*).tw,kf. 105273

17 (chatgpt* or gpt* or chat gpt* or chatbot* or chat bot or chat bots).tw,kf. 15884

18 (Claude or Gemini or Bard or Copilot or Quillbot or Jasper or "Dall E").tw,kf. 6839

19 (computer heuristic* or fuzzy logic).tw,kf. 3106

20 7 or 8 or 9 or 10 or 11 or 12 or 13 or 14 or 15 or 16 or 17 or 18 or 19 507894

21 exp Rehabilitation/ or Motor Skills/ or exp Physical Therapy Modalities/ 413410

22 (rehab* or re habilitation or re hab or habilitation).tw,kf. 257541

23 ((musculo* or msk or muscular or behavior* or behaviour* or neuro* or motor or cognitive or brain or sensory) and function*).tw,kf. 1212477

24 ((physical or exercise or motion or muscle or musculoskeletal or occupational or recreation or mobility or mobili?ation or neurodevelopmental or neuro developmental or coordination) adj3 therap*).tw,kf. 80131

25 ((endurance or muscle or plyometric* or resistance or movement or strength or weight* or mobility or mobili?ation or musculoskeletal or orthopaedic or orthopedic or gross motor or fine motor or gait or balance or neurodevelopmental or neuro developmental or coordination) adj3 (training or exercise*)).tw,kf. 81338

26 ((chiropractic or osteopathic or spinal or orthopaedic or orthopedic) adj3 (manipulation* or adjustment*)).tw,kf. 3278

27 (physiatry or ergotherap* or physiotherap* or physio therap* or kinesiology or physical activit*).tw,kf. 220218

28 (constraint induced movement therapy or CIMT).tw,kf. 5384

29 or/21-28 1970825

30 6 and 20 and 29 3801

| Database | Embase |
| --- | --- |
| Platform | Ovid |
| Limits | None |
| Date | June 9, 2025 |
| Results | 4971 |

<https://proxy.library.mcgill.ca/login?url=https://ovidsp.ovid.com/ovidweb.cgi?T=JS&NEWS=N&PAGE=main&SHAREDSEARCHID=4kCumHCHBV7UNPXvc6Ody8ivZqj0TblBEZlhkyviP6RVclGbmSU58QOzP46zOLmD0>

Embase Classic+Embase <1947 to 2025 Week 23>

1 exp child/ 3886864

2 exp pediatrics/ 151934

3 (child* or preschool or pre school or school aged or teen* or adolescen* or "young adult*" or youth* or toddler*).tw,kf,jw. 3177893

4 (pediatric* or paediatric*).tw,kf,jw. 1453633

5 1 or 2 or 3 or 4 5301235

6 1 or 2 or 3 or 4 or 5 5301235

7 exp artificial intelligence/ 144277

8 (artificial intelligence or AI or comput* intelligence or comput* reasoning or comput* vision system* or comput* knowledge or machine intelligence or intelligence system* or intelligent system*).tw,kf. 156018

9 (machine learning or transfer learning).tw,kf. 180019

10 (dimensionality reduction* or sing* value decomposition* or linear discriminant analys*).tw,kf. 22104

11 (ensemble learning* or ensemble model aggregation* or federated learning* or reinforcement learning* or predictive learning* or representation learning* or supervised learning* or semi supervised learning* or unsupervised learning* or multiple instance learning* or support vector machine*).tw,kf. 68026

12 (sentiment analys* or sentiment classification* or opinion mining).tw,kf. 2244

13 (particle swarm or swarm intelligence or classical PSO or classical PSOs or PSO TVAC).tw,kf. 5738

14 (machine pattern* or trend analys* or machine predict*).tw,kf. 11223

15 (Computational Neural Network* or Connectionist Model* or neural network* or computer neural network* or perceptron*).tw,kf. 158427

16 (deep learning or hierarchical learning or autoencoder* or large language model* or natural language processing*).tw,kf. 119107

17 (chatgpt* or gpt* or chat gpt* or chatbot* or chat bot or chat bots).tw,kf. 19756

18 (Claude or Gemini or Bard or Copilot or Quillbot or Jasper or "Dall E").tw,kf. 11364

19 (computer heuristic* or fuzzy logic).tw,kf. 3734

20 or/7-19 599796

21 exp rehabilitation/ or exp physiotherapy/ or exp physical performance/ 824777

22 (rehab* or re habilitation or re hab or habilitation).tw,kf. 394305

23 ((musculo* or msk or muscular or behavior* or behaviour* or neuro* or motor or cognitive or brain or sensory) and function*).tw,kf. 1656849

24 ((physical or exercise or motion or muscle or musculoskeletal or occupational or recreation or mobility or mobili?ation or neurodevelopmental or neuro developmental or coordination) adj3 therap*).tw,kf. 124008

25 ((endurance or muscle or plyometric* or resistance or movement or strength or weight* or mobility or mobili?ation or musculoskeletal or orthopaedic or orthopedic or gross motor or fine motor or gait or balance or neurodevelopmental or neuro developmental or coordination) adj3 (training or exercise*)).tw,kf. 115104

26 ((chiropractic or osteopathic or spinal or orthopaedic or orthopedic) adj3 (manipulation* or adjustment*)).tw,kf. 4143

27 (physiatry or ergotherap* or physiotherap* or physio therap* or kinesiology or physical activit*).tw,kf. 332489

28 (constraint induced movement therapy or CIMT).tw,kf. 9958

29 or/21-28 2817726

30 6 and 20 and 29 4971

| Database | CINAHL |
| --- | --- |
| Platform | Ebsco |
| Limits | None |
| Date | June 9, 2025 |
| Results | 736 |

https://proxy.library.mcgill.ca/login?url=https://search.ebscohost.com/login.aspx?direct=true&db=rzh&bquery=(((MH+%26quot%3bChild%2b%26quot%3b))+OR+((MH+%26quot%3bPediatrics%2b%26quot%3b))+OR+((MH+%26quot%3bAdolescence%2b%26quot%3b))+OR+((TI+(child*+OR+preschool+OR+pre+school+OR+school+aged+OR+teen*+OR+adolescen*+OR+%26quot%3byoung+adult*%26quot%3b+OR+youth*+OR+toddler*+OR+pediatric*+OR+paediatric*))+OR+(AB+(child*+OR+preschool+OR+pre+school+OR+school+aged+OR+teen*+OR+adolescen*+OR+%26quot%3byoung+adult*%26quot%3b+OR+youth*+OR+toddler*+OR+pediatric*+OR+paediatric*))))+AND+(((MH+%26quot%3bRehabilitation%2b%26quot%3b))+OR+((MH+%26quot%3bMotor+Skills%2b%26quot%3b))+OR+((TI+(rehab*+OR+re+habilitation+OR+%26quot%3bre+hab%26quot%3b+OR+habilitation+OR+physiatry+OR+ergotherap*+OR+physiotherap*+OR+physio+therap*+OR+kinesiology+OR+physical+activit*+OR+constraint+induced+movement+therapy+OR+CIMT))+OR+(AB+(rehab*+OR+re+habilitation+OR+%26quot%3bre+hab%26quot%3b+OR+habilitation+OR+physiatry+OR+ergotherap*+OR+physiotherap*+OR+physio+therap*+OR+kinesiology+OR+physical+activit*+OR+constraint+induced+movement+therapy+OR+CIMT)))+OR+((TI+(((physical+OR+exercise+OR+motion+OR+muscle+OR+musculoskeletal+OR+occupational+OR+recreation+OR+mobility+OR+mobili%3fation+OR+neurodevelopmental+OR+neuro+developmental+OR+coordination)+N3+therap*)))+OR+(AB+(((physical+OR+exercise+OR+motion+OR+muscle+OR+musculoskeletal+OR+occupational+OR+recreation+OR+mobility+OR+mobili%3fation+OR+neurodevelopmental+OR+neuro+developmental+OR+coordination)+N3+therap*))))+OR+((TI+(((endurance+OR+muscle+OR+plyometric*+OR+resistance+OR+movement+OR+strength+OR+weight*+OR+mobility+OR+mobili%3fation+OR+musculoskeletal+OR+orthopaedic+OR+orthopedic+OR+gross+motor+OR+fine+motor+OR+gait+OR+balance+OR+neurodevelopmental+OR+neuro+developmental+OR+coordination)+N3+(training+OR+exercise*))))+OR+(AB+(((endurance+OR+muscle+OR+plyometric*+OR+resistance+OR+movement+OR+strength+OR+weight*+OR+mobility+OR+mobili%3fation+OR+musculoskeletal+OR+orthopaedic+OR+orthopedic+OR+gross+motor+OR+fine+motor+OR+gait+OR+balance+OR+neurodevelopmental+OR+neuro+developmental+OR+coordination)+N3+(training+OR+exercise*)))))+OR+((TI+(((chiropractic+OR+osteopathic+OR+spinal+OR+orthopaedic+OR+orthopedic)+N3+(manipulation*+OR+adjustment*))))+OR+(AB+(((chiropractic+OR+osteopathic+OR+spinal+OR+orthopaedic+OR+orthopedic)+N3+(manipulation*+OR+adjustment*)))))+OR+((TI+(musculo*+OR+msk+OR+muscular+OR+behavior*+OR+behaviour*+OR+neuro*+OR+motor+OR+cognitive+OR+brain+OR+sensory)+AND+function*)+OR+(AB+(musculo*+OR+msk+OR+muscular+OR+behavior*+OR+behaviour*+OR+neuro*+OR+motor+OR+cognitive+OR+brain+OR+sensory)+AND+function*)))+AND+((((MH+%26quot%3bArtificial+Intelligence%2b%26quot%3b))+NOT+((MH+%26quot%3bRobotics%2b%26quot%3b)))+OR+((TI+(artificial+intelligence+OR+AI+OR+comput*+intelligence+OR+comput*+reasoning+OR+comput*+vision+system*+OR+comput*+knowledge+OR+machine+intelligence+OR+intelligence+system*+OR+intelligent+system*+OR+machine+learning+OR+transfer+learning+OR+dimensionality+reduction*+OR+sing*+value+decomposition*+OR+linear+discriminant+analys*+OR+ensemble+learning*+OR+ensemble+model+aggregation*+OR+federated+learning*+OR+reinforcement+learning*+OR+predictive+learning*+OR+representation+learning*+OR+supervised+learning*+OR+semi+supervised+learning*+OR+unsupervised+learning*+OR+multiple+instance+learning*+OR+support+vector+machine*+OR+sentiment+analys*+OR+sentiment+classification*+OR+opinion+mining+OR+particle+swarm+OR+swarm+intelligence+OR+classical+PSO+OR+classical+PSOs+OR+PSO+TVAC+OR+machine+pattern*+OR+trend+analys*+OR+machine+predict*+OR+Computational+Neural+Network*+OR+Connectionist+Model*+OR+neural+network*+OR+computer+neural+network*+OR+perceptron*+OR+deep+learning+OR+hierarchical+learning+OR+autoencoder*+OR+large+language+model*+OR+natural+language+processing*+OR+chatgpt*+OR+gpt*+OR+chat+gpt*+OR+chatbot*+OR+chat+bot+OR+chat+bots+OR+Claude+OR+Gemini+OR+Bard+OR+Copilot+OR+Quillbot+OR+Jasper+OR+%26quot%3bDall+E%26quot%3b+OR+computer+heuristic*+OR+fuzzy+logic))+OR+(AB+(artificial+intelligence+OR+AI+OR+comput*+intelligence+OR+comput*+reasoning+OR+comput*+vision+system*+OR+comput*+knowledge+OR+machine+intelligence+OR+intelligence+system*+OR+intelligent+system*+OR+machine+learning+OR+transfer+learning+OR+dimensionality+reduction*+OR+sing*+value+decomposition*+OR+linear+discriminant+analys*+OR+ensemble+learning*+OR+ensemble+model+aggregation*+OR+federated+learning*+OR+reinforcement+learning*+OR+predictive+learning*+OR+representation+learning*+OR+supervised+learning*+OR+semi+supervised+learning*+OR+unsupervised+learning*+OR+multiple+instance+learning*+OR+support+vector+machine*+OR+sentiment+analys*+OR+sentiment+classification*+OR+opinion+mining+OR+particle+swarm+OR+swarm+intelligence+OR+classical+PSO+OR+classical+PSOs+OR+PSO+TVAC+OR+machine+pattern*+OR+trend+analys*+OR+machine+predict*+OR+Computational+Neural+Network*+OR+Connectionist+Model*+OR+neural+network*+OR+computer+neural+network*+OR+perceptron*+OR+deep+learning+OR+hierarchical+learning+OR+autoencoder*+OR+large+language+model*+OR+natural+language+processing*+OR+chatgpt*+OR+gpt*+OR+chat+gpt*+OR+chatbot*+OR+chat+bot+OR+chat+bots+OR+Claude+OR+Gemini+OR+Bard+OR+Copilot+OR+Quillbot+OR+Jasper+OR+%26quot%3bDall+E%26quot%3b+OR+computer+heuristic*+OR+fuzzy+logic))))&type=1&searchMode=Standard&scope=site

| S19 | S5 AND S17 AND S18 | 736 |
| --- | --- | --- |
| S18 | S8 OR S9 | 75,431 |
| S17 | S10 OR S11 OR S12 OR S13 OR S14 OR S15 OR S16 | 709,403 |
| S16 | TI ( musculo* or msk or muscular or behavior* or behaviour* or neuro* or motor or cognitive or brain or sensory) and function* ) OR AB ( musculo* or msk or muscular or behavior* or behaviour* or neuro* or motor or cognitive or brain or sensory) and function* ) | 190,166 |
| S15 | TI ( ((chiropractic or osteopathic or spinal or orthopaedic or orthopedic) n3 (manipulation* or adjustment*)) ) OR AB ( ((chiropractic or osteopathic or spinal or orthopaedic or orthopedic) n3 (manipulation* or adjustment*)) ) | Display |
| S14 | TI ( ((endurance or muscle or plyometric* or resistance or movement or strength or weight* or mobility or mobili?ation or musculoskeletal or orthopaedic or orthopedic or gross motor or fine motor or gait or balance or neurodevelopmental or neuro developmental or coordination) n3 (training or exercise*)) ) OR AB ( ((endurance or muscle or plyometric* or resistance or movement or strength or weight* or mobility or mobili?ation or musculoskeletal or orthopaedic or orthopedic or gross motor or fine motor or gait or balance or neurodevelopmental or neuro developmental or coordination) n3 (training or exercise*)) ) | Display |
| S13 | TI ( ((physical or exercise or motion or muscle or musculoskeletal or occupational or recreation or mobility or mobili?ation or neurodevelopmental or neuro developmental or coordination) n3 therap*) ) OR AB ( ((physical or exercise or motion or muscle or musculoskeletal or occupational or recreation or mobility or mobili?ation or neurodevelopmental or neuro developmental or coordination) n3 therap*) ) | Display |
| S12 | TI ( rehab* or re habilitation or "re hab" or habilitation or physiatry or ergotherap* or physiotherap* or physio therap* or kinesiology or physical activit* or constraint induced movement therapy or CIMT ) OR AB ( rehab* or re habilitation or "re hab" or habilitation or physiatry or ergotherap* or physiotherap* or physio therap* or kinesiology or physical activit* or constraint induced movement therapy or CIMT ) | Display |
| S11 | (MH "Motor Skills+") | Display |
| S10 | (MH "Rehabilitation+") | Display |
| S9 | TI ( artificial intelligence or AI or comput* intelligence or comput* reasoning or comput* vision system* or comput* knowledge or machine intelligence or intelligence system* or intelligent system* or machine learning or transfer learning or dimensionality reduction* or sing* value decomposition* or linear discriminant analys* or ensemble learning* or ensemble model aggregation* or federated learning* or reinforcement learning* or predictive learning* or representation learning* or supervised learning* or semi supervised learning* or unsupervised learning* or multiple instance learning* or support vector machine* or sentiment analys* or sentiment classification* or opinion mining or particle swarm or swarm intelligence or classical PSO or classical PSOs or PSO TVAC or machine pattern* or trend analys* or machine predict* or Computational Neural Network* or Connectionist Model* or neural network* or computer neural network* or perceptron* or deep learning or hierarchical learning or autoencoder* or large language model* or natural language processing* or chatgpt* or gpt* or chat gpt* or chatbot* or chat bot or chat bots or Claude or Gemini or Bard or Copilot or Quillbot or Jasper or "Dall E" or computer heuristic* or fuzzy logic ) OR AB ( artificial intelligence or AI or comput* intelligence or comput* reasoning or comput* vision system* or comput* knowledge or machine intelligence or intelligence system* or intelligent system* or machine learning or transfer learning or dimensionality reduction* or sing* value decomposition* or linear discriminant analys* or ensemble learning* or ensemble model aggregation* or federated learning* or reinforcement learning* or predictive learning* or representation learning* or supervised learning* or semi supervised learning* or unsupervised learning* or multiple instance learning* or support vector machine* or sentiment analys* or sentiment classification* or opinion mining or particle swarm or swarm intelligence or classical PSO or classical PSOs or PSO TVAC or machine pattern* or trend analys* or machine predict* or Computational Neural Network* or Connectionist Model* or neural network* or computer neural network* or perceptron* or deep learning or hierarchical learning or autoencoder* or large language model* or natural language processing* or chatgpt* or gpt* or chat gpt* or chatbot* or chat bot or chat bots or Claude or Gemini or Bard or Copilot or Quillbot or Jasper or "Dall E" or computer heuristic* or fuzzy logic ) | Display |
| S8 | S6 NOT S7 | Display |
| S7 | (MH "Robotics+") | Display |
| S6 | (MH "Artificial Intelligence+") | Display |
| S5 | S1 OR S2 OR S3 OR S4 | Display |
| S4 | TI ( child* or preschool or pre school or school aged or teen* or adolescen* or "young adult*" or youth* or toddler* or pediatric* or paediatric* ) OR AB ( child* or preschool or pre school or school aged or teen* or adolescen* or "young adult*" or youth* or toddler* or pediatric* or paediatric* ) | Display |
| S3 | (MH "Adolescence+") | Display |
| S2 | (MH "Pediatrics+") | Display |
| S1 | (MH "Child+") | Display |

| Database | IEEE Xplore |
| --- | --- |
| Platform | N/A |
| Limits | None |
| Date | June 9, 2025 |
| Results | 1862 |

((((All Metadata:child*) OR (All Metadata:teen*) OR (All Metadata:pediatric*) OR (All Metadata:paediatric*) OR (All Metadata:adolescen*))) AND ((All Metadata:gait) OR (All Metadata:balance) OR (All Metadata:muscular ) OR (All Metadata:musculoskeletal) OR (All Metadata:rehabilitation) OR (All Metadata:physical therapy) OR (All Metadata:physical activity) OR (All Metadata:mobility) OR (All Metadata:movement*))) AND ((All Metadata:artificial intelligence) OR (All Metadata:ai) OR (All Metadata:computer intelligence) OR (All Metadata:machine learning) OR (All Metadata:machine pattern) OR (All Metadata:deep learning) OR (All Metadata:gpt))
